# A273 A PILOT PROSPECITVE, MULTI-CENTRE, RANDOMIZED CONTROLLED TRIAL COMPARING FECAL MICROBIOTA TRANSPLANTATION (FMT) TO PLACEBO AT INDUCING REMISSION IN MILD TO MODERATE CROHN'S DISEASE

**DOI:** 10.1093/jcag/gwad061.273

**Published:** 2024-02-14

**Authors:** K Wong, D Kao, H Jijon, R Franz, N Narula, F Peerani, C Turbide, K Kroeker, P Moayyedi, K Madsen

**Affiliations:** University of Alberta, Edmonton, AB, Canada; University of Alberta, Edmonton, AB, Canada; University of Calgary, Calgary, AB, Canada; University of Alberta, Edmonton, AB, Canada; McMaster University, Hamilton, ON, Canada; University of Alberta, Edmonton, AB, Canada; University of Calgary, Calgary, AB, Canada; University of Alberta, Edmonton, AB, Canada; McMaster University, Hamilton, ON, Canada; University of Alberta, Edmonton, AB, Canada

## Abstract

**Background:**

FMT has shown promise at inducing remission in ulcerative colitis. The effect of FMT on inducing remission in Crohn’s disease however remains unknown.

**Aims:**

To evaluate the efficacy of FMT at inducing remission in mild-to-moderate Crohn’s disease

**Methods:**

This double-blind, randomized, trial conducted at 3 Canadian academic centers (Edmonton, Hamilton and Calgary) randomized. Adult patients with mild-to-moderate Crohn’s disease with modified Harvey Bradshaw Index (mHBI) score of ampersand:003E5 and at least one objective measure of inflammation (CRP ≥ 8mg/L or fecal calprotectin (FCP) ≥ 250 ug/g) on stable dosing of concomitant therapy were assigned to FMT or placebo. First assigned treatment was by colonoscopy followed by 20 [DK1] capsules weekly for 7 weeks. Endoscopic severity (SES-CD) was assessed at baseline during colonoscopic delivery of assigned treatment and at week 8 by a blinded central reader using video recording. The primary endpoint was the proportion of patients in both clinical (mHBI ampersand:003C 5) and endoscopic remission (SES-CD ≤ 5) at week 8. Responders (reduction of HBI ≥ 3 or HBI ≤ 5 and reduction of CRP or FCP by ≥ 10%) in FMT group were eligible to continue with open-label FMT capsule every 2 weeks. Non-responders in the placebo group were permitted to restart the trial at week 0 with open-label FMT. Secondary outcomes included patient reported outcomes and safety outcomes.

**Results:**

Between July 2017 to Jun 2021, 28 were randomized (16 to FMT and 12 to placebo). The study team voluntarily suspended the trial twice due to Health Canada safety warnings regarding potential transmission of multi-drug resistant organisms and SARS-CoV-2 through FMT. At week 8, 0% (0/16) of patients in the FMT group versus 8.3% (1/12) in the placebo group reached the primary endpoint of combined clinical and endoscopic remission. Of the 50% (8/16) in the FMT group who achieved a clinical response and continued on open-label FMT every 2 weeks, 12.5% (1/8) achieved the primary endpoint. Of the 7 non-responders in the placebo group who restarted the trial at week 0 and received open-label FMT, 28.6% (2/7) achieved primary endpoint at week 8 on open-label FMT. No death or hospitalization were observed. The study was terminated early at the recommendation of the Data Safety Monitoring Board.

**Conclusions:**

FMT was not effective at inducing remission in Crohn’s disease using the FMT regimen in this study. However, this study was limited by a small sample size and recruitment barrier during the COVID pandemic. Future studies may consider other strategies to potentially enhance treatment response, including antibiotic pre-treatment, optimized donor-recipient pairing, and concomitant anti-inflammatory diet.

**Funding Agencies:**

CIHR

